# An integrated approach to mental health and disaster preparedness: a cluster comparison with earthquake affected communities in Nepal

**DOI:** 10.1186/s12888-018-1863-z

**Published:** 2018-09-15

**Authors:** Courtney Welton-Mitchell, Leah Emily James, Shree Niwas Khanal, Alexander Scott James

**Affiliations:** 1 Institute of Behavioral Science, Natural Hazards Center, University of Colorado-Boulder, 483 UCB, Boulder, CO 80309-0483 USA; 2 Transcultural Psychosocial Organization – Nepal, GPO Box 8974/CPC Box 612, Baluwatar, Kathmandu, Nepal

**Keywords:** Mental health, Disaster, Preparedness, Intervention, Nepal, Earthquake, Aftershocks

## Abstract

**Background:**

On 25th April 2015, Nepal experienced a 7.8 magnitude earthquake, followed by countless aftershocks. Nearly 9000 people were killed and over 600,000 homes destroyed. Given the high frequency of earthquake and other natural hazards in Nepal, disaster preparedness is crucial. However, evidence suggests that some people exposed to prior disasters do not engage in risk reduction, even when they receive training and have adequate resources. Mental health symptoms, including those associated with prior disaster exposure, may influence engagement in preparedness. Perceived preparedness for future disasters may in turn influence mental health. Social cohesion may influence both mental health and preparedness.

**Methods:**

We developed and tested a hybrid mental health and disaster preparedness intervention in two earthquake-affected communities in Nepal (*N* = 240), about 2.5 months after the April 25th, 2015 earthquake. The 3-day intervention was culturally adapted, facilitated by trained Nepalese clinicians and focused on enhancing disaster preparedness, mental health, and community cohesion. Communities were selected based on earthquake impacts and matched on demographic variables. The intervention was administered initially to one community, followed by the other receiving the intervention shortly thereafter. Survey data was collected across three time points. Focus groups were also conducted to examine intervention impact.

**Results:**

At pre-intervention baseline, greater depression symptoms and lower social cohesion were associated with less disaster preparedness. Depression and PTSD were associated with lower social cohesion. Participation in the intervention increased disaster preparedness, decreased depression- and PTSD-related symptoms, and increased social cohesion. Mediation models indicated that the effect of intervention on depression was partially explained by preparedness. The effect of the intervention on disaster preparedness was partially explained by social cohesion, and the effect of intervention on depression and on PTSD was also partially explained by social cohesion. Data from focus groups illuminate participant perspectives on components of the intervention associated with preparedness, mental health and social cohesion.

**Conclusions:**

This mental health integrated disaster preparedness intervention is effective in enhancing resilience among earthquake-affected communities in Nepal. This brief, cost-effective group intervention has the potential to be scaled up for use with other communities vulnerable to earthquakes and other natural hazards.

**Trial registration:**

Clinical Trials Registry-India, National Institute of Medical Statistics. Registration number: CTRI/2018/02/011688. http://ctri.nic.in/Clinicaltrials/login.php Retrospectively registered February 5th, 2018. First participant enrolled July 2015.

## Background

On 25th April, 2015, Nepal experienced a 7.8 magnitude earthquake, followed by countless aftershocks, including one of 7.3 magnitude on 12th May, 2015. The earthquake affected 14 out of the 75 districts of the country, with just under 9000 people killed and over 600,000 homes destroyed [1]. In the aftermath, many survivors were forced to live in inadequate temporary dwellings, with limited access to water and food, due to the impact of the earthquake on water supplies and agriculture-based livelihoods [1]. The situation was exacerbated by monsoon rains, resulting in associated flooding and landslides in communities struggling to recover from the earthquake [1–3].

Unfortunately, such events are not unusual in Nepal. Approximately 10,000 families are affected each year as a result of natural hazards in Nepal [4]. In the three decades prior to the 2015 earthquake, nearly 80 events have been recorded that have killed over 11,000 people and affected more than 5 million [4]. This environmental vulnerability is compounded by a decade long civil war, challenges in governance, and a lack of public trust in elected officials [5–7].

Considering such challenges in Nepal, and other countries facing similar natural hazards, it is important to understand factors contributing to recovery, and influencing engagement in preparedness for future disasters. Even in resource-poor settings such as Nepal, low cost disaster preparedness strategies can be implemented, including identifying safe and risky areas in the community; planning associated evacuation routes; storing documents safely; stockpiling water, food and some other basic supplies; reinforcing dwellings, and more. Yet, despite substantial efforts to train disaster-prone communities in Nepal and elsewhere in such risk reduction strategies, growing evidence suggests that often people do not engage in preparedness, even when they possess sufficient resources, receive training, and/or have a history of disaster exposure [8–11].

There is some evidence to suggest that reasons for this may in part be psychological in nature and dependent on social context [7, 12–15]. Risk perception literature indicates that preparedness behaviors are likely linked to perceived risk of severity and personal vulnerability, ability to prepare (efficacy), and likelihood that specific behaviors will actually mitigate risk [16, 17]. Associated research underscores the importance of considering demographic variables, including types of vulnerability, when examining engagement in preparedness. Studies indicate that some individuals, such as those with physical and mental challenges, the elderly and the homeless, may face particular barriers to preparedness [18, 19]. Despite a rich and increasingly complex literature about factors potentially linked to disaster preparedness, there is a scarcity of research examining the potential role of mental health symptoms in preparedness.

There are several reasons to believe that mental health symptoms may influence disaster preparedness. Posttraumatic stress, anxiety, depression, and somatic symptoms are typically found in communities experiencing disasters [20]. Common forms of distress indicated by rapid assessments conducted weeks after the earthquake in Nepal include fear, anxiety, sadness, anger, sleep difficulties, and increased risk of suicide [21]. Some of those experiencing distress may struggle with related challenges for years, interfering with their ability to work and fulfill family responsibilities. In a meta-analysis of disaster survivors in 80 countries, nearly one-quarter of those reporting symptoms also experienced substantial functional impairment, limiting their ability to perform daily activities [22]. Such mental health symptoms may do more than impede recovery; they may also impact one’s ability to engage in future disaster preparedness [18].

Although overall distress may interfere with preparedness, it may actually be the case that specific symptoms undermine preparedness. Hopelessness and helplessness, often associated with depression, may interfere with motivation to prepare, linked to a belief that the worst will occur regardless of one’s efforts [13]. Avoidance symptoms associated with PTSD (e.g., attempts to avoid memories, places, and other reminders of traumatic experiences) may explain avoidance of disaster-related content, including thoughts or behaviors associated with future disasters [23]. Given this, it is possible that disaster preparedness interventions may be more effective when they also address disaster-related mental health symptoms [23, 24]. Interventions can address feelings of hopelessness and limited efficacy and teach participants skills to engage with, rather than avoid, potentially anxiety-inducing disaster preparedness content.

The relationship between preparedness and mental health may be bidirectional. Just as improved mental health may encourage increased preparedness, preparedness behaviors may in turn positively impact mental health. Feeling adequately prepared may increase efficacy and decrease feelings of anxiety and hopelessness [25]. Given this, disaster mental health interventions may also benefit from inclusion of disaster preparedness content.

In countries such as Nepal, it may be beneficial to focus on preparedness and mental health in a group setting, underscoring the value of collective action to address concerns associated with natural hazards. Community or social cohesion has been defined as the ability to work together to problem solve and is assumed to involve a sense of trust and belonging, a willingness to participate in the community and to help other community members [26]. Social cohesion may play an especially important role in motivating collective action to address threats, including natural hazards, in post-conflict settings such as Nepal [7, 27].

There is also a rich literature on the link between social support and mental health across the lifespan, including in times of adversity [28]. The strength of a community’s social networks and the extent to which it operates cohesively may affect the wellbeing of individual community members, as well as the ability of the entire community to address natural hazards through coordinated efforts [29–34]. However, disasters may disrupt social networks, exceeding the ability of individuals and the community to cope, interfering with support, and undermining trust [35, 36]. Group interventions enhancing social cohesion and encouraging peer-based help-seeking and help-giving, may encourage resilience in communities facing natural hazards. This may be particularly true of disaster preparedness and wellbeing related peer-support in communities at risk of recurring events.

### Current study

Little is known about what type of disaster mental health interventions are effective in the months following an earthquake in settings such as Nepal. Given the chronic disaster-prone context, it is critical that communities be given the tools necessary to prepare for future natural hazards and to recover when disasters do occur. With this in mind, we developed and evaluated a 3-day mental health integrated disaster preparedness intervention for earthquake survivors in Nepal. The community-based group intervention is culturally adapted, incorporates coping skills and community building activities, and was tested using a cluster comparison design. We hypothesized the following: disaster preparedness, mental health (depression and PTSD), and social cohesion will all be significantly associated at Time 1 (baseline, prior to the intervention). Specifically, disaster preparedness will be associated with mental health symptoms (depression and PTSD) and social cohesion, such that greater symptoms and lower social cohesion are linked to less preparedness. Social cohesion will be associated with mental health symptoms, such that higher rates of depression and PTSD are associated with lower social cohesion. Participation in the 3-day intervention will result in: an increase in disaster preparedness; a decrease in mental health symptoms (depression, PTSD); and an increase in social cohesion. Finally, mediation of the intervention’s effects is anticipated such that, a change in mental health symptoms will partially explain the impact of the intervention on preparedness, a change in preparedness will partially explain the intervention’s impact on mental health; and a change in social cohesion will partially explain the impact of the intervention on preparedness and on mental health.

## Methods

### Participants and procedures

The research was conducted between June and September 2015, following the 25th April earthquake in Nepal. Bhaktapur is among the districts hardest hit by the earthquake, with 333 dead, 2101 injured, nearly 20,000 homes fully damaged, and another almost 10,000 houses partially damaged [37]. Two communities in Bhaktapur district, Nepal were selected for the project based on review of local government data, including damage reports, and visits to communities by members of the research team: 1) Changunarayan Municipality, Ward no. 2; Chhaling area and 2) Mahamanjushree Municipality, ward no. 3; Tathali (Sudol and Nemaphuki) area. Based on information provided by the District Administration Office in Bhaktapur, Changunarayan and Mahamanjushree municipalities reported the highest levels of infrastructural damage and casualties within the district. Within these municipalities, specific subareas or wards were chosen based on: (1) available information about earthquake damage, (2) consultations with community leaders about needs and interest in intervention, and (3) demographic variables including ethnicity and socioeconomic status, in order to select two similar communities, as necessitated by the research design. Of note, no community was excluded due to lack of interest by community leaders; all communities indicated interest in the intervention. As a result, the two communities with the greatest damage and most similar demographics were selected for this research.

Household lists representing all dwellings within the affected areas were developed in collaboration with community leaders and included some home visits for confirmation that the dwelling was occupied. In Chhaling (Community A), 196 households were identified in the sampling frame, and in Tathali (Community B), 180 households were identified. Of these, 250 households were assessed for eligibility, using a recruitment script, and approached in the order encountered starting from the area with the greatest earthquake damage in each community. Researchers approached any type of dwelling as many community members within the sample frame were residing in ad hoc temporary dwellings at the time data was collected. One adult within each household was eligible to participate (age 18–65; household decision-maker, gender balanced; available to attend 3-day intervention training); no additional inclusion or exclusion criteria were utilized beyond ability to consent. A total of 240 individuals were selected to participate in the research interview and the 3-day intervention (one person was excluded due to age and at nine households, individuals were not available due to work or childcare commitments).

Participants received drinks and snacks as compensation, consistent with compensation offered in similar settings in Nepal. Intervention participants received meals, local travel compensation, materials (printed documents, note-taking materials), and a “disaster supply kit” (including bucket, water bottle, flashlight, whistle, bandages, dust mask, plastic sleeves for documents) for a total cost of no more than $15 per participant.

### Design

Research utilized a quasi-experimental design that allowed for rigorous assessment of effectiveness. Specifically, a stepped-wedge design [38] was employed. Each of two clusters received the intervention within a few weeks, with the first community to receive the intervention selected at random. This design enabled both clusters to receive the intervention as soon as capacity allowed and avoided individual-level randomization and assignment to control group which may have been poorly received and potentially ethically inappropriate in the post-earthquake context in Nepal.

All participants (240 total) completed brief (30 min) assessment interviews at Time 1 (mid-July) prior to participating in the 3-day intervention workshops. After the intervention was administered in one community (Chhaling), a second assessment using the same interview schedule was completed for all participants in both communities (Time 2; approximately two weeks after the first assessment). Next, after the second community (Tathali) received the intervention, a third assessment was conducted for all participants (Time 3; approximately 2 weeks after the second assessment). All interviews took place from early July through end-August 2015. Focus groups were conducted in each community about 2 weeks after the intervention. There were two sub-groups of research team members: those conducting interviews and those administering the intervention. Allocations to condition were not shared with those conducting interviews.

The 3-day mental health integrated disaster preparedness intervention was implemented in six intervention groups of 20 participants each; three groups were run concurrently in each community. Groups were facilitated by six Nepali clinicians (two leading each group), familiar with the specific subcultural groups and fluent in the local languages. All Nepali clinicians had between 2 and 6 years of community leadership experience. Their educational backgrounds ranged from a 6 month-certificate in counseling to a three-year Master’s degree in Psychology. Two additional support staff with health degrees were hired to assist. Facilitators were trained over the course of 2 weeks by senior members of the research team, including the second author, a doctoral level social worker/psychologist, who also provided onsite supervision during implementation. Clinicians did not conduct any of the associated interviews at time 1, 2 or 3.

The intervention used in this research was based on a model initially developed for use with earthquake survivors in Haiti (James L, Welton-Mitchell C, Noel JR, James A: Integrating mental health and disaster preparedness in intervention: a randomized controlled trial with earthquake and flood-affected communities in Haiti, submitted), and subsequently modified for use with flood survivors in Nepal. The version of the manualized intervention used in this study was adapted by the research team for use with earthquake survivors in Bhaktapur, Nepal through a series of consultations and workshops with local Nepali clinicians, and other staff from the partner organization, several of whom were from communities that were culturally and linguistically similar to project communities. Additional adaptations were made based on facilitator suggestions during the training process. The adaptation process included review of all manualized intervention content for cultural compatibility and comprehension. Examples of additions to the manual included culturally-specific stories (e.g., a story about a mouse swimming in yogurt commonly told to Nepali children was used to send a message about hope), discussions about karmic beliefs linked to disaster attributions and mental health stigma, and the use of cultural symbols to help to create a sense of safety. As a part of the curriculum, facilitators were trained to introduce topics associated with cultural and religious belief systems, encouraging participants to engage in lively yet supportive discussions. This approach to intervention development and associated cultural adaptation is consistent with best practice guidelines and recommendations from others working in Nepal [39, 40].

The mental health integrated disaster preparedness intervention utilizes an experiential approach to delivering session content. Participants engage in facilitated discussion and sharing of personal experiences, providing peer-support throughout the process. In addition, they acquire and practice coping skills targeting disaster-related distress. Hands-on training is provided in disaster preparedness and peer-based mental health models for use by participants in their own lives and to support other community members. The content of the sessions is based on a standardized manual [41], publicly available online in English and Nepali, and described in detail in other publications [42].

In order to examine intervention impact, focus groups were conducted in both communities about 2 weeks after the intervention. Participants included a random selection of 1) men who participated in the intervention; 2) women who participated in the intervention; 3) a mixed gender group of participants identified as having mental health concerns (highest levels of PTSD and depression at T1); and 4) family members of participants. Although eight FGDs were planned, only seven were conducted as family members were not available in one location due to agricultural work responsibilities. In total, data was collected from 58 persons (37 participants in Chhaling and 21 participants in Tathali).

### Measures

Instruments were selected based on research conducted previously in Nepal by this research team and others, including several instruments validated specifically for use in this context. Trained Nepali researchers conducted participant interviews in Nepali (with supplemental Newari as needed during a few interviews). Data were collected using Qualtrics survey software on handheld tablets. The survey instrument was intentionally brief to alleviate potential burden on participants, given the post-earthquake circumstances. A subset of variables, constituting primary outcomes, are examined in this manuscript.

**Demographic** questions included *age, marital status, children, religion, caste/ethnicity, employment, education and length of time living in the community.*

Exposure to **chronic stressors** in the post-earthquake environment was assessed at baseline using three items adapted from the Humanitarian Emergency Settings Perceived Needs (HESPER) [43]. Participants could respond with yes or no to the following questions: *Do you have a serious problem: (1) because you do not have enough water that is safe for drinking, cooking, or bathing, or enough food, or good enough food, or because you are not able to cook food?* (2) *because you do not have enough income, money or resources to live?* (3) *with your physical health? For example, because you have a physical illness, injury or disability.*

**Earthquake exposure** was assessed at baseline through six investigator-developed items appropriate for the context, such as *was your house badly damaged or destroyed as a result of the earthquake/aftershocks?* (with yes/no response options).

Self-reported **disaster preparedness** was measured at all timepoints using a 7-item investigator developed checklist in which participants were asked to indicate which behaviors they had engaged in to prepare for future disasters: *made a disaster supply kit; stored extra food or water for animals; put important documents in a safe place; secured dwelling/made stronger in some way; modified furniture in some way (secured, raised); discussed a family evacuation plan or where to meet up if separated; considered safe and vulnerable places in your community.* Items in checklists were developed through focus groups and interviews conducted by the research team over the course of two previous studies [23, manuscript in preparation]. Sums of selected items were calculated for each participant. The potential for bias associated with self-report was minimized by having researchers ask to see evidence of preparedness during household interviews. Internal consistency for this sample was adequate (Cronbach’s alpha = 0.59, reported at T1 for this and other measures).

**Depression symptoms** were assessed using a version of the 9-item Patient Health Questionnaire (PHQ-9) [44], that had been previously translated and adapted for use in Nepal by TPO-Nepal [45]. Questions are framed in terms of, *Over the past two weeks how often have you been bothered by any of the following problems?* Responses range from *not at all* (0) to *nearly every day* (3). The measure has demonstrated good psychometric properties when used previously in Nepal [45]. Internal consistency for this sample was good (Cronbach’s alpha = 0.81).

**Posttraumatic Stress symptoms** were assessed using the 17-item Posttraumatic Stress disorder (PTSD) Checklist – Civilian Version (PCL-C) [46], previously translated and adapted for use in Nepal by TPO-Nepal. Responses focus on *how much difficulty/discomfort you had* in the past week associated with specific symptoms, linked to a prior stressful event. Response options range from not at all (1) to extremely (5). The scale has demonstrated good psychometric properties in this context [47, 48]. Internal consistency for this sample was good (Cronbach’s alpha = 0.89).

**Social cohesion** was assessed using two items: *People in this community are willing to help their neighbors* and *People in this community generally don’t get along with each other* - reverse coded. Response options ranged from 1 = *strongly disagree* to 5 = *strongly agree* [49] (*r* = 0.25, acceptable for scales with few items measuring broad characteristics; [49]).

**Help-seeking** regarding mental health and disaster preparedness/response was assessed using two investigator-developed items: *Would you be comfortable seeking help from others if you were struck by sadness or mental tension that made your life difficult?* [50] and *Would you be comfortable seeking help from others if you needed something to prepare for or in the aftermath of a disaster?* Responses options on a 4-point scale ranged from 1 = *I would not be comfortable at all*, to 4 = *I would be very comfortable.*

#### Focus groups

Focus groups included several questions, primarily related to stressors and reactions to the intervention; questions highlighted in this manuscript: *What are the primary stressors in your life right now?* and *What did you learn from the intervention?*

### Data analysis

Analyses were conducted using R and STATA 13. We assessed the comparability of the two communities (matching on demographic, chronic stress and earthquake exposure variables) with t-tests, assuming equal variance, except when Levine’s test indicated otherwise. Fisher’s exact test and χ2 of contingency tables were used for categorical demographic data.

Demographic analyses included all Time 1 data; for main intervention and mediation analyses, subjects who participated in the intervention and for whom data at Time points 2 and/or 3 were available (in addition to time point 1) were included. Intent to treat analyses [51], wherein subjects who did not complete the intervention training were nevertheless included in their as-randomized (community-level) treatment group, were also conducted. Here, no requirement for follow-up data being present at Time 2 or Time 3 in addition to Time 1 was enforced (i.e., all data available was analyzed).

PTSD and depression scale responses were averaged and subjects’ data were included in analyses if at least two-thirds of scale items were available. When a whole scale’s data was missing – mainly caused by loss of the subject’s entire data at a time point due to attrition – no such mean imputation was used. In a few other cases, 1 or 2 items from the scale were missing. For PTSD and depression scales where means were analyzed, the scale’s value was taken as the mean of the available items. Of note, results were consistent when data was examined using both as-treated and intent-to-treat approaches to analyses, suggesting that the impact of missing data due to attrition or noncompliance on results may have been minimal. Responses on other scales were summed, and if a subject was missing data for any scale item the subject’s data for that time point was excluded.

For the purpose of intervention-related analyses, conventional approaches for stepped-wedge controlled randomized-to-community designs were used [52]. Participants were clustered within communities resulting in a three-level hierarchical mixed effects model (measurements across time clustered within participants clustered within community) with fixed effects of time point and intervention and random intercepts at community and participant level. This approach permits distinguishing of intervention effects from intervention-independent time-related trends. In some cases, no third-level clustering (random intercept) at the community level was included, when it was determined that the variance component did not improve model fit, using likelihood ratio testing.

Scale measures were approximately Gaussian distributed and therefore linear modeling was employed, single-item help-seeking questions were ordinal variables and accordingly analyzed using a cumulative logit-link model. Treatment effect coefficients (and associated standard errors as well as *p*-values) represent the difference in measured scales between control and intervention conditions. Control data comes from both baseline datasets (Time 1) as well as Tathali at Time 2. Intervention data comes from Chhaling at Time 2 and Chhaling and Tathali at Time 3.

To understand the manner in which the intervention affected mental health and disaster preparedness, mediation analyses were conducted in a structural equations modeling framework, with clustering of measurements across time within subject accounted for by a latent multilevel effect. Bias-corrected confidence intervals for indirect effects were calculated by bootstrapping as recommended [53] using 5000 resamples. These analyses incorporated data from all three time points and controlled for time-related trends. Mental health measures and disaster preparedness were explored as both mediators and outcomes; social cohesion was explored as a mediator. Of note, ‘intervention’ was a binary 0/1 dummy variable coding whether the participant had received the intervention yet or not, in an identical manner to that of the main intervention effect analyses, extended to the SEM framework for these analyses. Time/assessment point was also controlled for separately in the models.

Focus group data was intended to further describe the context in which data collection took place and to illuminate intervention effects. With this objective in mind, following the quantitative data analysis, one coder reviewed translated focus group transcripts for common themes in response to the question, *what are the primary stressors in your life right now?* This bottom up process resulted in the following categories being used to sort representative quotes: *ongoing hazards/threats; distress; social division/disintegration; lack of basic resources*. The same process was followed when coding responses to the question, *what did you learn from the intervention?* resulting in the following categories: *disaster preparedness; managing distress (coping); willingness to provide mental health support; willingness to seek mental health support; and social cohesion/collaboration (working together to solve challenges).*

## Results

### Participant retention

**Fig. 1:**
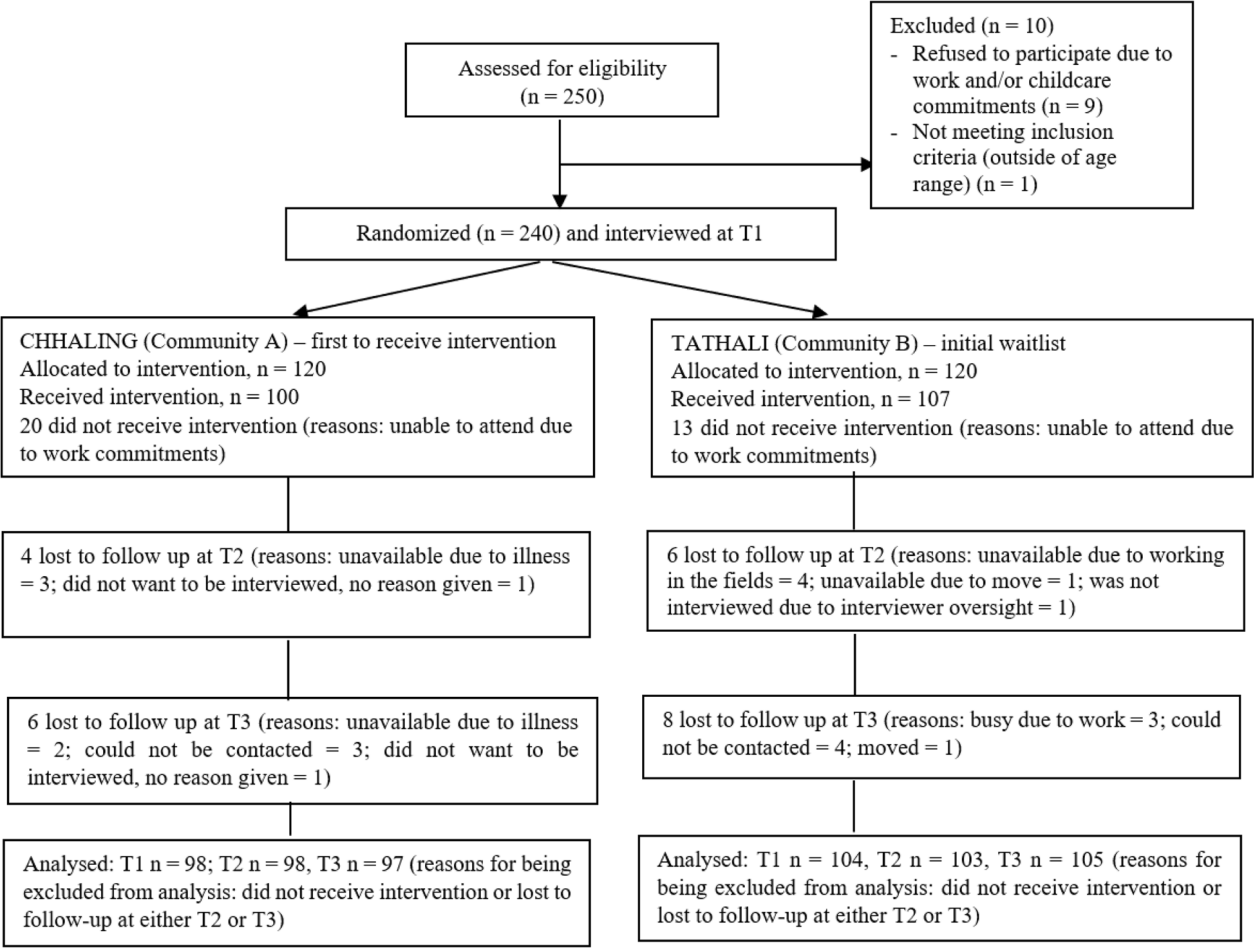
Participant flow diagram. Analyses conducted based on as-treated, with outcomes examined for those who completed the intervention as allocated. Results and corresponding conclusions were similar when analyzed as intent-to-treat [53]

Figure 1 summarizes the flow of participants. Although 240 participants were interviewed at baseline (Time 1) and allocated to the intervention in the two communities, 207 received the intervention (the majority who could not attend had work commitments). At Time 2, 10 participants were lost to follow-up, and at Time 3, 14 participants were lost. See Fig. 1 for details.

### Baseline characteristics

Demographic data, collected at T1, are presented in Table 1. Sixty percent of participants were female, and the mean age was 38 years. Most participants were married with two children. Almost all participants were Hindu, and communities were of mixed Brahmin/Chettri and Janajati (primarily Newar) ethnicity. Close to half worked in agriculture, and a quarter worked in the home. A fifth of the sample were unable to read or write, with over half completing only secondary education. More than 90% had lived in the communities for more than 10 years at the time of the study. Everyone in the sample experienced the earthquake in April 2015, with over 90% having their home badly damaged as a result. Of those, over 70% had to relocate to a temporary structure outside their home. Approximately a third of the sample struggled to meet basic needs, including experiencing difficulties with access to food and water.

**Table 1:** Demographics including chronic stressors and earthquake exposure at Time 1

Variable	Total sample	Chhaling (Community A)	Tathali (Community B)
Total participants	240	120	120
Gender	60% female; 40% male	61% female; 39% male	59% female; 41% male
Age	M = 38 years, Range 18–72	M = 40 years, Range 18–72	M = 37 years, Range 18–68
Marital status	86% married	90% married	82% married
Children	M = 2.1, Median: 2.0	M = 2.2, Median: 2.0	M = 1.9, Median: 2.0
Religion	99% Hindu	99% Hindu; 1% Other	99% Hindu; 1% Christian
Caste/ethnicity	65% Janajati (primarily Newar); 34% Brahmin/Chettri; 1% Dalit/Other	87% Janjati (primarily Newar); 13% Brahmin/Chettri	42% Janajati (primarily Newar); 55% Brahmin/Chettri 1%; Dalit; 2% other
Employment	50% agriculture; 24% work at home; 9% professional; 9% student; 5% business/labor	53% agriculture; 26% work at home; 6% professional; 6% student; 4% business/labor	46% agriculture; 22% work at home; 12% professional; 12% student; 5% business/labor
Education	20% unable to read or write; 61% primary to secondary; 19% intermediate or above	20% unable to read or write; 63% primary to secondary; 17% intermediate or above	19% unable to read or write; 60% primary to secondary; 21% intermediate or above
Time in community	90% more than 10 years	88% more than 10 years	91% more than 10 years
Chronic Stressors	*% with yes response (serious problem because….)*		
Insufficient water or food	30%	36%	24%
Insufficient income for basic necessities	28%	31%	25%
Poor health *(physical illness, injury or disability)*	17%	13%	21%
Earthquake exposure			
Experienced April 25th, 2015 earthquake	100%	100%	100%
House badly damaged or destroyed due to earthquake	92%	94%	89%
If yes, sleeping in a temporary structure outside	72%	66%	78%

Communities were matched on demographic variables, chronic stressors and earthquake exposure at T1. Only significant difference in demographics between communities at T1 was for caste/ethnicity, with significantly more Janajati in Chhaling and more Brahmin/Chettri in Tathali

The two communities appear well matched on demographic variables, chronic stressors, and earthquake exposure at T1 (see Table 1). The only variable demonstrating significant difference across communities was caste/ethnicity. There were significantly more Janajati in Chhaling and more Brahmin/Chattri in Tathali (χ^2^ = 54.046, df = 3, *p* < 0. 0001). There were no differences between communities in baseline mental health characteristics; however, social cohesion was lower in Chhaling than Tathali (*p* = 0.02). This difference should not affect interpretation of analysis results as each subject acts as its own control in the approach used, and both communities ultimately receive the intervention, which is incorporated into intervention effect coefficients.

### Relationships among variables

At Time 1, pre-intervention baseline, disaster preparedness was associated with mental health symptoms and social cohesion, such that greater depression symptoms and lower social cohesion were associated with less preparedness; PTSD symptoms were not associated with preparedness. Social cohesion was also associated with mental health symptoms, such that higher rates of depression and PTSD were associated with lower social cohesion (see Table 2).

**Table 2:** Correlations between variables at baseline

Measure	Disaster prepared-ness	Depression	PTSD	Social cohesion	Help-seeking, mental health- related	Help-seeking, disaster preparedness-related
Disaster preparedness	–	0.14*	−0.06	0.14*	0.00	0.09
Depression	−0.14*	–	0.73***	−0.22***	0.01	−0.15*
PTSD	−0.06	0.73***	–	−0.22***	0.04	−0.07
Social cohesion	0.14*	−0.22***	−0.22***	–	0.17**	0.19**
Help-seeking, mental health-related	0.00	0.01	0.04	0.17**	–	0.55***
Help-seeking, disaster preparedness-related	0.09	−0.15*	−0.07	0.19**	0.55***	–

*** *p* < 0.001, ** *p* < 0.01, * *p* < 0.05

### Intervention effects

Exploration of intervention effects revealed that participation increased disaster preparedness, decreased depression and PTSD-related symptoms, and increased social cohesion. Within-subject contrasts between Time 1 and Time 3 for these measures within the Chhaling community which received the intervention directly after T1 were also significant, suggestive of an effect lasting for at least some weeks after the intervention. Mental health-related and disaster-related help-seeking intention also increased as a result of intervention participation (Time 1 to Time 3 contrasts within Chhaling for these measures were not significant) (Table 3).

**Table 3:** Comparison of changes over time, intervention effects

Variable	Unstandardized intervention coefficient (standard error)	Effect size (Cohen’s D)	Within subject contrast T1 to T2 for Chhaling community Estimate (SE)	Within subject contrast T1 to T3 for Chhaling community Estimate (SE)
Disaster preparedness	0.75*** (0.18)	0.49	1.19*** (0.15)	1.57*** (0.15)
Depression (PHQ)	−0.26*** (0.06)	0.49	−0.38*** (0.05)	− 0.35*** (0.05)
PTSD (PCL-C)	−0.27*** (0.06)	0.39	−0.33*** (0.05)	− 0.19*** (0.05)
Social cohesion	0.80** (0.28)	0.40	0.83*** (0.24)	0.54* (0.24)
Help-seeking, mental health-related	0.76* (0.30)	–	0.56* (0.27)	0.36 (0.26)
Help-seeking, disaster preparedness-related	0.69* (0.31)	–	0.63* (0.27)	0.366 (0.26)

*** *p* < 0.001, ** *p* < 0.01, * *p* < 0.05

All results presented here are as-treated analyses; in all cases, results and corresponding conclusions were similar when analyzed as intent-to-treat [51]. Because participants were given a disaster supply kit (see methods), a separate analysis was conducted wherein “made a disaster supply kit” and “important documents in a safe place” items were excluded from the disaster preparedness scale; results were similar.

### Mediation model results

**Fig. 2:**
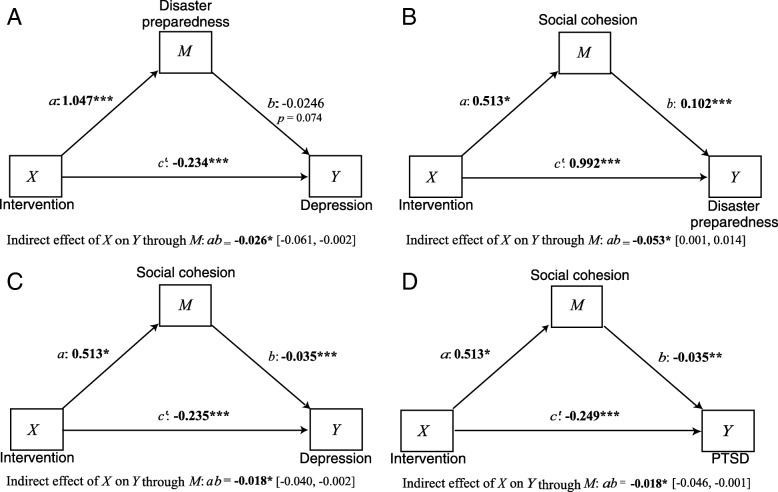
Mediation diagrams. **a** effect of intervention on depression is partially explained by preparedness; (**b**) effect of intervention on disaster preparedness is partially explained by social cohesion; (**c**) effect of intervention on depression is partially explained by social cohesion; (**d**) effect of intervention on PTSD is partially explained by social cohesion. In each model, two equations were used: 1) the effect of the intervention on the mediator (*a* path), and 2) the effects of the mediator on the outcome variable (*b* path) and the intervention on the outcome variable *(c’* path). The direct effect of the intervention on outcomes is given by *c’* and the mediated or indirect effect of the intervention is given by the product *ab*

Mediation models indicated that the effect of intervention on depression is partially explained by its effects on preparedness. The effect of intervention on disaster preparedness is partially explained by social cohesion, and the effect of intervention on depression and on PTSD is also partially explained by social cohesion (see Fig. 2).

In a similar model exploring whether the intervention indirectly affected PTSD symptoms by affecting preparedness there was no significant mediated effect (ab = 0.0123, 95% conf. interval [− 0.170, 0.050]). This is consistent with the failure to find a correlation between PTSD symptoms and preparedness at time 1. Two related models assessed whether the intervention effect on preparedness was mediated by its influence on mental health symptoms. Neither depression (ab = 0.049, 95% conf. interval [− 0.019, 0.117]) nor PTSD (ab = − 0.016, 95% conf. interval [− 0.068, 0.046]) were statistically significant mediators. In all models, a significant direct effect of the intervention was found, consistent with main intervention effect analyses.

### Focus groups

Data from focus groups further illuminates participant perspectives on stressors at the time of the study and components of the intervention associated with preparedness, mental health and social cohesion. In response to the question, *what are the primary stressors in your life currently?* primary themes were: *ongoing hazards/threat, distress, social division/disintegration, and lack of basic resources*. In response to the question, *what did you learn from the intervention?* Primary themes were: *disaster preparedness, coping skills to manage distress, willingness to provide mental health support (to other community members), willingness to seek mental health support, and the importance of working together to solve challenges* Representative quotes are presented in Table 4.

**Table 4:** Participant response to focus group questions (7 focus groups, *n* = 58)

Theme	Representative quotes
Response to - *What are the primary stressors in your life right now?*	
Ongoing hazards/threats	We have felt hundreds of aftershocks after the earthquake./Thundering and lightning also scared us very much./There are different kind of disaster and flooding and landslide along with earthquake./This is the season of monsoon./ Even the temporary shelter is about to collapse./There is destruction everywhere.
Distress	People still do have fear and stress./ Restlessness, it’s difficult to sleep, children do not even want to sleep./ We are very much scared./ I feel that the ground is shaking again./ We have been trying to forget everything, but we keep on dreaming about it./ Just spent all day crying without eating./ Irritated for small reasons./ We do not have any interest to work, could not even laugh./ We are dependent on alcohol to minimize stress./ Children will not go to school, even parents are avoiding their job, thinking that they are going to die soon.
Social division/disintegration	After the earthquake fighting and quarrelling has been increasing in the society./ Most of the children and women were affected because of this fighting./ Those who did not get relief materials were angry. People who are clever get relief materials and those who are not didn’t get any./ People are fighting about land./ After the earthquake occurred, people are scattered, living apart from relatives and friends, this might be the reason for stress./ These days, there are high chances of robbery and theft.
Lack of basic resources	After the earthquake, most of the houses are collapsed and all of our needs were increased but there were not enough resources, no money and no source of income./ Parents are not able to fulfill the basic needs of their children./ We don’t have work so we don’t have money./ Our field was washed away./ Our economic condition is very poor so how we would build house again./ We need to sell our land in a cheap price./ We have problem of drinking water./ Crops will last for 2–3 months and after that we will not have food.
Response to - *What did you learn from the intervention?*	
Disaster preparedness	Now we know how to protect our lives and belongings during the disaster, so we are very much relieved./ After taking the training, all the responsibilities in the family are divided and we have already packed our belongings, important documents are kept safely, medicines are kept and stored some rice, dal, wheat and flour safely in a drum./ We realize that we need to prepare necessary materials in a bag so we can just grab it./ We have learned to share information with family, friends and community about disaster preparedness.
Coping skills to manage distress	After joining this training, irritation and fright has decreased./ We learned some techniques to get free from mental stress - breathing exercise, meditation./ We were very much scared and disturbed by the earthquake, but this training has helped us to come out from this fear. It has brought peace in our lives./ Different kind of exercise for different kind of thoughts. If we are having difficulties in sleeping then can do body relaxation exercise./ After the training we were feeling light./ I used to get very angry and after I learned that exercise, I am being able to control my anger./ My stress level has decreased./ We must try to cope instead of avoiding.
Willingness to provide mental health support	We are helping each other, providing emotional support./ I have taught my family and children how to minimize stress by doing exercises./ I have taught my brother a technique to control his anger./ If people are suffering or stressed because of disaster, because his house collapsed, we can console them./ To have feelings of helping others is useful./ If people are having problems we need to hear their problems. Before we used to neglect them but now, we have a feeling to help and support them.
Willingness to seek mental health support	Even we have stress, fear and anxiety, we should not keep it to ourselves, and we have to share it with others, so that we could feel lighter./ When I have restlessness and uncertain thoughts then I share it with my friends./ [During the training] we were able to share our thoughts about heart-mind related problems.
Importance of working together to solve challenges	The training has taught us to be cooperative, to help each other and to maintain good relationship with the society./ Unity is strength. If we work together in the society it will be easier to cope up with a natural disaster like an earthquake./ If an elephant gets separated from his herd a lion can attack him but if he is in the group no one can touch him./ We should maintain good relationship, be cooperative./ Before we used to think why do we need to help, but now we learn that we need to save others as well. So without depending on any other organization or government, we can do better if we work together, help each other.

## Discussion

In a disaster-prone context such as Nepal, is it critical to develop and test community-based interventions that prioritize prevention and expedite recovery. Previous research highlights potential connections between disaster preparedness, mental health, and social cohesion, suggesting that these areas should be a focus of interventions. We developed and tested just such an intervention, unique in that it integrates mental health and preparedness components within a single model designed to encourage community cohesion, including peer-based help-giving and help-seeking.

We hypothesized that disaster preparedness, mental health (symptoms of depression and PTSD), and social cohesion would be significantly associated at baseline. As predicted, at Time 1, greater depression symptoms and lower social cohesion were associated with less preparedness. PTSD was not however, associated with preparedness. Depression and PTSD were both associated with lower social cohesion. Post-earthquake, pre-intervention associations provide support for the idea that interventions providing mental health and coping supports, and emphasizing community cohesion activities, may have an impact on preparedness.

Data from focus groups further underscores the value of such an intervention. There were numerous difficulties facing communities at the time of this research, a few months after the initial earthquake. Communities were experiencing regular aftershocks and the onset of monsoon, with many living in flimsy temporary dwellings. Community members reported fear and sleep difficulties associated with aftershocks. Some were feeling hopeless and overwhelmed, crying frequently, having difficulties eating, feeling irritable, and lacking the motivation to engage in daily tasks. Social division also appeared to be on the rise in the aftermath of the earthquake. Participants suggested that theft was a problem, and that fighting was becoming more common, including conflicts related to the distribution of relief materials and access to land. Social networks appear to have been disrupted, with people in some areas forced to live apart from friends and relatives and having to form new social bonds.

The intervention appears to have been effective in addressing several of these concerns. The impact of the intervention was examined using an analysis approach that permits distinguishing of intervention effects from intervention-independent time-related trends. As hypothesized, participation in the intervention increased disaster preparedness, decreased depression and PTSD related symptoms, and increased social cohesion. Mental health and disaster-related help-seeking also increased. These results point to the value of a brief, group-based, mental health integrated disaster preparedness intervention, administered a few months after an acute event such as the earthquake.

To further examine the underlying theoretical model, mediation analyses were performed. Mediation was anticipated such that, a change in mental health symptoms would partially explain the impact of the intervention on preparedness, a change in preparedness would partially explain the impact of the intervention on mental health, and a change in social cohesion would partially explain the impact of the intervention on preparedness and on mental health. In all models, a significant direct effect of the intervention was found, consistent with main intervention effect analyses. In addition, models indicated that the effect of intervention on depression was partially explained by preparedness. However, no mediated effect on PTSD symptoms was found, consistent with the lack of correlation between PTSD symptoms and preparedness at baseline. In addition, the effect of the intervention on preparedness was not mediated by its influence on mental health symptoms. This is somewhat surprising, as in related work with earthquake and flood survivors in Haiti we found that the effect of the intervention on preparedness was mediated by mental health symptoms (PTSD, depression, and anxiety) (James L, Welton-Mitchell C, Noel JR, James A: Integrating mental health and disaster preparedness in intervention: a randomized controlled trial with earthquake and flood-affected communities in Haiti, submitted). Future research should examine these relationships further to determine to what extent culture and context may play a role in mental health symptoms potentially interfering with engagement in preparedness.

Although neither depression nor PTSD were statistically significant mediators of the effect of the intervention on preparedness, results suggest that preparedness behaviors may positively impact mental health (symptoms of depression). These results support the premise that being given the knowledge and encouragement to engage in low cost, easy to implement preparedness, can increase efficacy and alleviate feelings typically associated with depression [25].

In addition, the effect of the intervention on disaster preparedness is partially explained by social cohesion, and the effect of intervention on mental health (depression and PTSD) is also partially explained by social cohesion. By encouraging social cohesion, or the ability to work together to problem solve and to help other community members [26], it is possible that the intervention strengthened the bonds that make collective preparedness and social support possible. Effects may have been strengthened by the intervention’s emphasis on group discussions, exercises, games, and disaster and mental health related help-giving and seeking roleplays. Findings are consistent with the rich literature on the relationship between social support and mental health [28]. Thus, an intervention which enhances social cohesion, and encourages peer-based help-seeking and help-giving, may be an important strategy for alleviating distress and encouraging preparedness.

Data from focus groups helps to clarify participant perspectives on components of the intervention associated with preparedness, mental health and social cohesion. Focus groups participants emphasized specific preparedness strategies that they were implementing as a result of the training including: sharing preparedness information with others in the community, division of family responsibilities during a disaster, safe storage of items (documents, medicine, food), and preparation of a ‘go-bag’. Participants also emphasized the acquisition of coping skills enabling them to help themselves and others, including skills to manage stress, fear, recurring thoughts, irritability/anger, sleep difficulties, and avoidance behaviors. Some participants also mentioned the peer support component, for example: *we are helping each other, providing emotional support; if people are having problems we need to hear their problems, before we used to neglect them but now, we have a feeling to help and support them.* Others spoke about a willingness to seek support from others: *even we have stress, fear and anxiety, we should not keep it to ourselves, and we have to share it with others, so that we could feel lighter.* This suggests that not only were participants able to utilize the skills from the training to support one another, they also recognized the potential benefits of seeking support from others. This is consistent with the increase in mental health and disaster related help-seeking at Time 2. Such emphasis on peer-based mental health and psychosocial support models is crucial in settings such as Nepal where mental health treatment is limited, especially for those in rural areas and with limited economic means [39].

Likewise, content from focus groups helps to elucidate how participants frame the construct of social cohesion, and its relationship to disaster preparedness. Many emphasized the importance of working together to solve challenges, especially in contexts in which help from outside may be limited: *The training has taught us to be cooperative, to help each other and to maintain good relationship with the society; If we work together in the society it will be easier to cope up with a natural disaster like an earthquake; Before we used to think why do we need to help, but now we learn that we need to save others as well; So without depending on any other organization or government, we can do better if we work together, help each other.* Previous research in Nepal suggests that communities affected by the 10-year civil war may be especially receptive to intervention frameworks emphasizing collective action to cope with threats [27].

### Limitations and strengths

Ongoing hazards made it difficult to access some communities in the aftermath of the 2015 earthquake in Nepal. Some areas in remote locations, and with greater earthquake impacts, were ruled out due to concerns over safety of the research team. Ideally such research could be conducted in more remote, less accessible areas, where communities are facing greater challenges in terms of mental health and preparedness, including limited access to formal mental health treatment.

Ethical concerns about random assignment to condition by household excluded the possibility of conducting a randomized controlled trial (RCT) so soon after the earthquake. Instead, a cluster comparison was utilized, matching communities on demographic variables and earthquake exposure, and resulting in only a brief waiting period before each of the two communities received the intervention. Future research should consider an RCT, with individual assignment to condition, comparing the intervention to ‘treatment as usual’ curriculum (both disaster preparedness as usual and mental health as usual) to isolate the mechanism of change, further elucidating the added value of combining mental health and disaster preparedness in one intervention.

The intervention was tested across three time points over a relatively short period. Future research could examine the impact of the intervention over a longer period, such as 6 to 12 months, in order to determine duration of effects. Optimal dose should also be considered to determine whether there is any added value in extending the duration of the 3-day intervention.

Despite these limitations, the results of this intervention were robust. The mental health integrated disaster preparedness intervention enhanced disaster preparedness, mental health and social cohesion in two earthquake-affected communities in Nepal. In addition, baseline associations between variables and mediation models provided some support for the theoretical framework and point to potential mechanisms of change.

## Conclusion

The UN and others have called for increased attention to preparedness efforts. Others have warned that extreme weather events fueled by climate change will have an increasing impact on mental health, requiring appropriate intervention [54]. Evidence gap reviews of health interventions in humanitarian crises have resulted in a call for low-cost, low-intensity (brief, non-invasive), group-based mental health interventions [55]. This study addresses this gap. The brief group-based intervention has the potential to be scaled up for use not only throughout Nepal, but in other countries experiencing earthquakes and other natural hazards.
